# Social media and orthodontics: Are our patients scrolling?

**DOI:** 10.1177/14653125211042025

**Published:** 2021-09-06

**Authors:** Nausheen Siddiqui, Matthew Chia, Mohammad Owaise Sharif

**Affiliations:** 1 Department of Orthodontics, University College London Hospitals Foundation Trust, Eastman Dental Hospital, London, UK; 2 Department of Orthodontics, Croydon University Hospital, Croydon, UK

**Keywords:** social media, orthodontics, compliance

## Abstract

**Objective:**

To assess patients’ awareness of the availability of information related to orthodontics on social media, and to explore patients’ willingness to engage with social media to aid with orthodontic treatment.

**Design:**

Prospective cross-sectional survey.

**Setting:**

This survey was conducted at Croydon University Hospital orthodontic department.

**Participants:**

A total of 125 orthodontic patients, both new and in treatment. There were no exclusion criteria.

**Methods:**

All participants completed a questionnaire designed to explore their awareness, access to and utilisation of social media as well as their willingness to engage with social media to support orthodontic treatment. There were no age restrictions or exclusion criteria.

**Results:**

Of the patients, 99% had access to social media. Of these patients, 64% were aware that social media platforms were available to help with orthodontic treatment, 30% had utilised social media related to orthodontics, with the most popular platforms being Instagram (n = 17) and Snapchat (n = 12). Of the patients, 73% stated that they would be willing to use social media in the future to support orthodontic treatment.

**Conclusion:**

Social media can be engaging, accessible and versatile, and has been shown to be effective at improving patients’ knowledge regarding orthodontic treatment. As such, it may be used as a valuable tool for information provision to engage orthodontic patients. Awareness of the availability of orthodontics content on social media is increasing; however, only one-third of participants had previously used it to aid with orthodontic treatment. Given the availability of information on social media targeted at orthodontic patients there is a need to assess the quality of this information and if appropriate navigate patients towards high-quality, effective resources.

## Introduction

Social media is a form of electronic communication through which users create and share content to participate in social networking. Over 200 well-known networking sites are available to share information, ideas, personal messages and other content, including videos. There are over 3.5 billion daily social media users worldwide and it has been reported that an average of three hours per day is spent by individuals on social media (Tjepkema, 2020). Social media is a platform that can allow treatment information to be delivered interactively in a variety of formats (visual, text, videos, etc.) that can be accessed at times accessible to individual patients globally, largely due to the proliferation of smartphones, tablets and computers, which allows the provision of versatile, readily available information. To this end, the availability of social media is having a significant impact in healthcare, and this provides an opportunity to engage with patients in non-traditional formats with a view to improving treatment outcomes. Of millennials, 90% are active social media users and this group now forms the majority of the orthodontic patient cohort (Emarketer, 2020). In 2015, 89% of orthodontic patients and parents reported using social media; it is highly likely that this figure will have increased since this report (Nelson et al., 2015).

In 2013, social media usage was examined in 130 orthodontic patients in a cross-sectional study (Henzell et al., 2013). The authors found that 6.7% of patients had considered using social media as a source of information provision. In 2019, interest in social media related to orthodontics had significantly increased with Sharif et al. (2019) reporting that 21% of orthodontic patients had sourced information related to orthodontic treatment on social media using YouTube, Facebook, blogs, Instagram, Twitter and Pinterest. Social media may have a positive impact on information retention and awareness. Al-Silwadi et al. (2015) and O’Brien and Duane (2017) reported that supplementing verbal and written information for patients in fixed appliances with YouTube videos improved their knowledge compared with patients who received information via traditional methods only. Sampson et al. (2021) reported that patients exposed to temporary anchorage devices on social media are more likely to accept them as a treatment option.

Further research in this area is important to enable orthodontists to ensure the methods of information provision and patient engagement are contemporary and patient-centred.

## Objective

To assess patients’ awareness of the availability of information related to orthodontics available on social media and their willingness to engage with social media to aid with orthodontic treatment.

## Design

This was a prospective cross-sectional survey.

## Setting

The survey was conducted at the orthodontic department of Croydon University Hospital. This a National Health Service district general hospital serving a population based in the South of London.

## Participants

A total of 125 orthodontic patients, both new and in treatment, were included. There were no exclusion criteria.

## Methods

**Figure 1.**
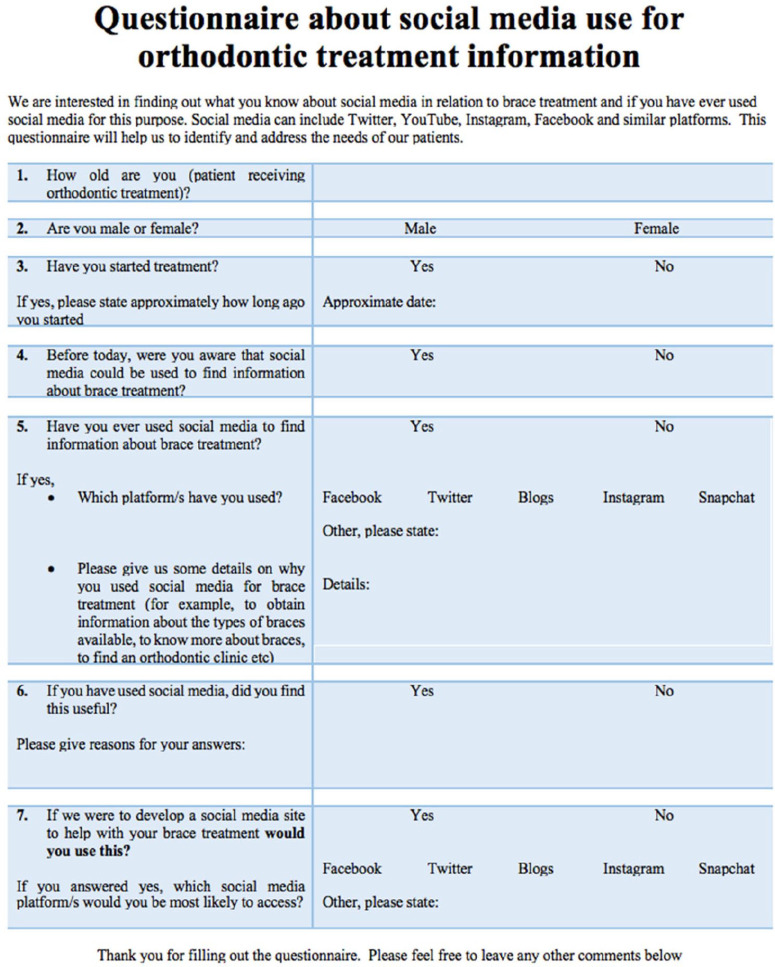
Data collection sheet.

Questionnaires were issued to 125 consecutive patients (new patients and in treatment) attending the Croydon University Hospital orthodontic department. A formal sample size calculation was not required as this was a survey; however, the sample size was based on a similar study conducted by Sharif et al. (2019). The participants were asked to complete a seven-item data collection form. The questionnaire was designed to explore patients’ awareness, access to and utilisation of social media to support orthodontic treatment (Figure 1). This is a modified version of the questionnaire used in the paper by Sharif et al. (2019). Furthermore, patients’ willingness to engage with and use social media to aid with orthodontic treatment was explored. All questionnaires were returned anonymously by posting the completed surveys into a concealed box. There were no age restrictions and parents of patients who were too young to complete the questionnaire independently were asked to complete the questionnaire with their children.

## Results

**Figure 2.**
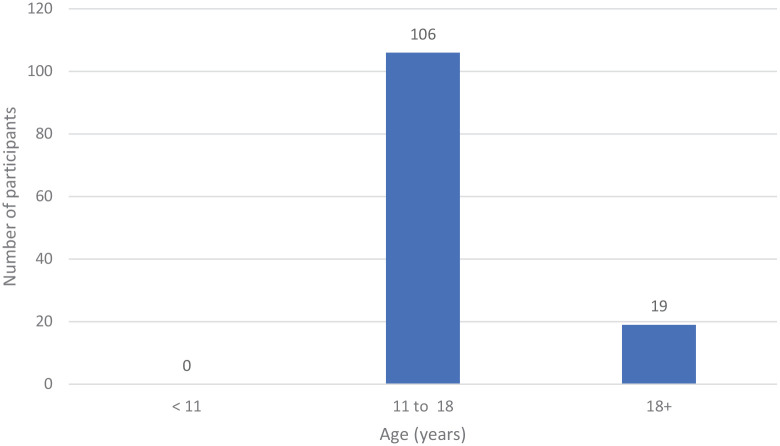
Age range of participants.

All data collection forms were returned and analysed using descriptive statistics. The age range of participants was 11–33 years; the majority (106 participants, 85%) were aged 11–18 years (Figure 2).

In total, 83 female patients and 42 male patients completed the data collection sheets; 78 patients were in treatment, whereas 47 patients had not yet started treatment. The majority (n = 80, 64%) were aware that social media platforms were available to find information about orthodontic treatment and 37 patients (30%) had utilised social media related to orthodontics.

**Figure 3.**
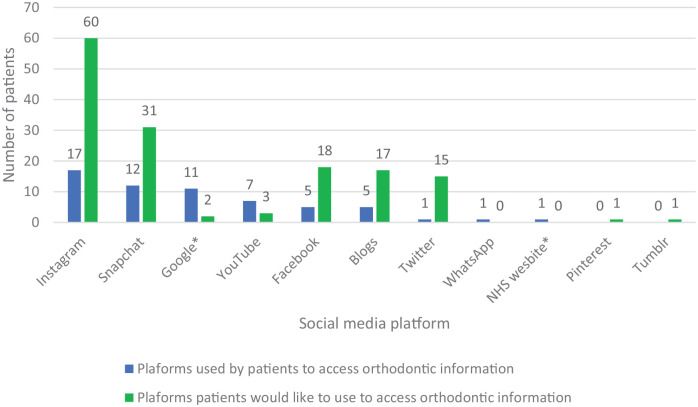
Platforms used by patients to access orthodontic information and platforms patients would like to use to access orthodontic information. *Platforms which are not social media networks.

Among the patients who had previously used social media to access orthodontic information, the most common reasons given were the following: to gain information on the outcomes of orthodontic treatment; types of appliances; and information regarding the practical aspects of treatment (Table 1). The most popular platforms used to access information about braces were Instagram (n = 17, 46%) followed by Snapchat (n = 12, 32%) (Figure 3).

**Table 1.** Comments from patients regarding reasons for using social media to source information.

‘Why I might need braces and what braces do’
‘Types of braces available’
‘To see what having braces would be like including the procedure of putting them on’
‘I searched ways of fixing an open bite’
‘How to deal with pain when I get it’
‘To see the results of braces’
‘Information from real people’
‘The duration of treatment’
‘To make sure my braces were 100% clean’
‘To choose colours’
‘Aftercare of braces’
‘Prepare for surgery’
‘Easiest place to access information’

Of those patients who had used social media, 31 found it useful; however, six patients did not find it useful. Comments from those who found social media useful can be seen in Table 2.

**Table 2.** Comments from participants who found social media useful.

‘How to treat my teeth and my braces, awareness of what to do and what not to do.’
‘It answered questions and gave me helpful solutions without having to call the hospital.’
‘Taught me how to brush properly.’
‘What products to use to clean my teeth.’
‘To get an idea of the end result.’
‘Videos and tips from patients and orthodontists explaining everything that happens with braces including information about the experience and how they are put on.’
‘It helped me make my decision.’
‘The NHS website was well informed and had appropriate information.’
‘Gave me an insight into my surgery.’

Comments provided by patients who did not find social media useful were as follows:

• ‘I did not know if what I read was what would happen if I did receive treatment.’
• ‘The accuracy of information cannot always be trusted – scientific journals are more trustworthy.’
• ‘There was not much information on the subject.’

Of the patients, 91 (73%) stated that they would be willing to use social media in the future to support their orthodontic treatment. Of these patients, the platforms that the participants would ideally like the information to be available on are shown in Figure 3, demonstrating that Instagram (n = 60, 66%) and Snapchat (n = 31, 34%) are the most popular.

## Discussion

This service evaluation demonstrates that among orthodontic patients there is awareness, utilisation and a willingness to engage with social media to support treatment. The majority of respondents were aged 11–18 years (85%); this is to be expected given that a large cohort of orthodontic patients fit this profile. Two-thirds of orthodontic patients reported being aware that social media was available to obtain information about orthodontic treatment; however, only one-third reported utilising social media for this purpose. This current study uses a similar population to the sample used by Sharif et al. (2019) and the results suggest that awareness of social media by patients in relation to orthodontics has increased from 23% to 34%. The present study was carried out before the COVID-19 pandemic, and it is likely that awareness of social media related to orthodontics will have further increased since the start of the pandemic. These findings support the notion that there is a need for the orthodontic profession to utilise social media to engage with the adolescent orthodontic population.

Instagram and Snapchat were the most popular platforms that patients reported using to research information about orthodontics. Both platforms allow access to short videos, less than 1 minute in duration, which are likely to be useful in captivating and holding the audiences’ attention. A cross-sectional study of 477 orthodontic patients carried out in Saudi Arabia also reported that Instagram was the favoured social media platform among patients aged 13–20 years (Alalawi et al., 2019); Twitter, Snapchat and YouTube were more popular in among patients aged 21–30 years, and WhatsApp was most frequently used by patients aged around 40 years. Interestingly, in addition to age, there were also differences in the preference of social media sites based on socioeconomic status, level of education and gender. No further information was reported on which platforms are preferred by different populations and why. It has, however, been reported that female patients use social media more than male patients as they like to keep close ties and gain social information.

Most patients using social media to gather information about orthodontic treatment reported that they found it useful. The main reasons that patients reported for sourcing information were to gain information on the outcomes of orthodontic treatment, types of appliances and information regarding the practical aspects of treatment. This is consistent with comments made by orthodontic patients on Twitter and Instagram, as found by Graf et al. (2020), where the researchers categorised 361 orthodontic related posts in a 30-day period. They also identified that there was a difference between the posts on different sites regarding the feelings towards orthodontic treatment. Both Papadimitrou et al. (2020) and Graf et al. (2020) reported that more posts were categorised as positive than negative, with the latter being the case for Instagram posts but not for Twitter. The difference between different posts on different sites may be a result of functional differences that lead patients to express themselves in specific ways, or it may be because different sites attract different demographics of patients. This leads us to the conclusion that as well as having different target audiences, different social media sites may have different uses rather than being a ‘one size fits all’ approach with YouTube delivering information to patients, whereas Snapchat and Twitter may allow content creation by patients and thereby making it easier for them to engage more with their experiences.

Despite the many potential advantages of social media to support treatment, including information provision, patient engagement and the ability to potentially improve patient outcomes, there are a number of potential challenges. First, there is a rapid proliferation of the availability of social media sites, making it increasingly difficult for patients and health professionals to identify high-quality information. Furthermore, there are limited published data relating to the quality of information content on orthodontic social media sites and networks. YouTube has been studied more than other social media platforms and the majority of the literature on the subject suggests that the quality of YouTube videos as a method of information provision in orthodontics is of a low standard. One of the reasons reported for this was that the videos assessed were largely created by patients rather than professionals (Da Silva et al., 2019; Knosel et al., 2011; Lena and Dindaroglu, 2017; Ustdal and Guney, 2020).

### Implications for future research and practice

The results of this paper largely reflect the views of adolescent patients in one region of the UK and therefore the generalisability of the results may be limited. Further research could explore willingness to use social media and the platforms in other age groups, i.e. adult orthodontic patients, as well as other regions of the UK. This information could be used to influence the development of social-media–based resources, including advertising and information provision, tailored to each demographic, for example, Instagram may most appropriate for engaging with adolescent patients whereas Twitter may be the desired platform to engage with adult patients.

As social media usage and the availability of social media targeted at orthodontic patients increases, there is a need to assess its quality. This should include an assessment of information accuracy and usability. This would then aid clinicians to navigate patients towards high-quality, effective resources. In the interim, individual clinicians should assess any social media sources they are considering recommending to patients in terms of quality and accuracy of information content, or it should have been approved by a reputable body, e.g. British Orthodontic Society.

## Conclusion

Although the awareness of social media to aid with orthodontic treatment is increasing, only one-third of the patients had previously utilised social media to obtain orthodontic-related information. Interestingly, three-quarters of patients reported they would be willing to use social media to aid with orthodontic treatment. Further research is required to assess information availability and quality across the different social media platforms. At present, clinicians should assess any social media sources they are considering recommending to patients in terms of quality and accuracy of information content.
